# Effect of Silicone Rubber of a Waste Composite Insulator on Cement Mortar Properties

**DOI:** 10.3390/ma12172796

**Published:** 2019-08-30

**Authors:** Gang Qin, Zhongshuo Shen, Yongqiang Yu, Lidan Fan, Hongwei Cao, Chaowei Yin

**Affiliations:** 1 School of Materials Science and Engineering, Henan Polytechnic University, Jiaozuo 454000, China; 2 Henan Key Laboratory of Underground Engineering Disaster Prevention, Henan Polytechnic University, Jiaozuo 454000, China; 3 International Joint Research Laboratory of Henan Province for Underground Space Development and Disaster Prevention, Henan Polytechnic University, Jiaozuo 454000, China; 4 Henan Province Engineering Laboratory for Eco-architecture and the Built Environment, Henan Polytechnic University, Jiaozuo 454000, China; 5 State Grid Henan Electric Power Research Institute, Zhengzhou 450052, China

**Keywords:** waste silicone rubber, composite insulator, cement mortar, thermal insulation, noise reduction

## Abstract

Due to the rapid growth of the electric power system, the silicone rubber composite insulator is so widely employed around the world. However, the aging damage and decommission of it inevitably generates plenty of waste silicone rubber. It takes up large amounts of land for non-degradation, which is becoming a serious environmental issue. In this paper, the effort has been made to reuse the silicone rubber of composite insulator by peeling, breaking and adding it into the cement mortar to partly replace river sand by 5%, 10%, 15%, 20% and 30% in volume. Moreover, the surface modification of silicone rubber particles with H_2_O_2_ and KOH is explored to improve hydrophilicity. The mechanical strength of silicone rubber mortar and modified silicone rubber mortar are measured in detail, and the effects of particle size, content and modification of silicone rubber on the properties of mortar are investigated. This study shows that at least a 5% addition of silicone rubber can reduce the strengths, while the 15% addition is obviously favorable for increasing the toughness. Furthermore, the mortar strength gets promoted due to the surface modification. Lastly, the superior thermal insulation and noise reduction of the mortar are obtained as the addition of silicone rubber particles, and an equation between dry apparent density and thermal conductivity of mortar has been developed.

## 1. Introduction

**Figure 1:**
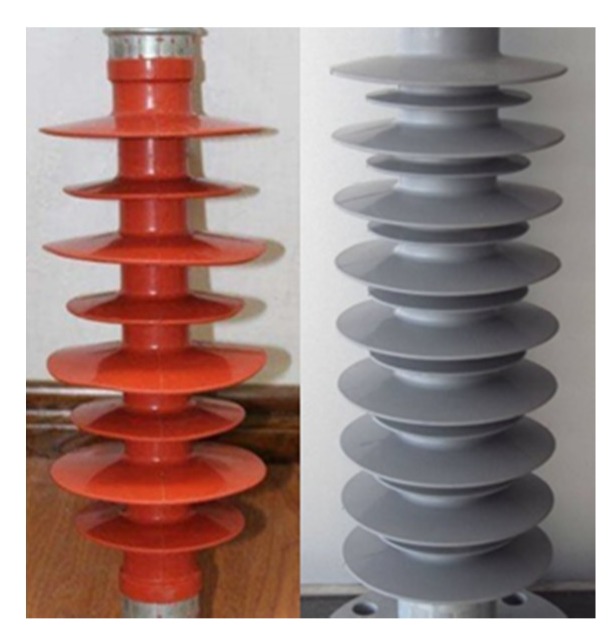
Silicone rubber based composite insulators.

**Figure 2:**
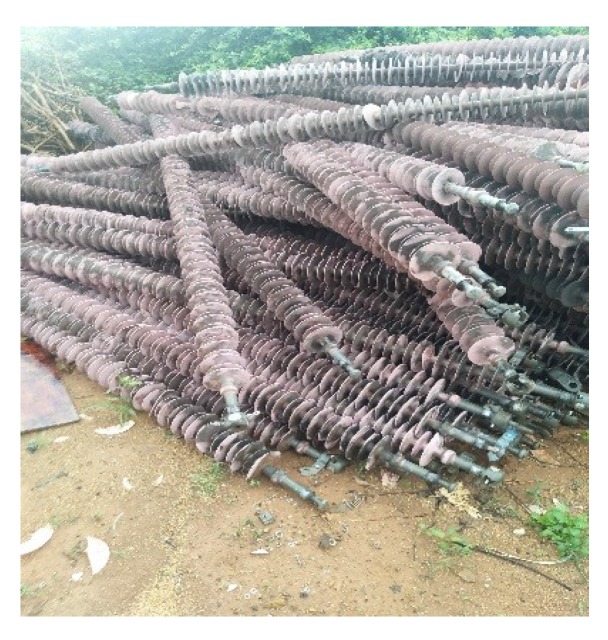
Landfill site of waste composite insulators.

The silicone rubber based composite insulators (Figure 1) have been widely used all over the world for ultra-high and extra-high voltage transmission lines in recent years [1,2,3]. In China, by the end of 2018, the statistical data from the State Grid Corporation of China shows that the number of composite insulators for more than 66 kV levels has exceeded 10 million. The composite insulators are aging gradually because of the long service time (10–15 years) and the natural deterioration due to the electric field, mechanical stress, atmospheric environment and thermal radiation [4]. The aged composite insulators cannot meet the requirements of electricity transmission, and will be discarded [5,6]. The cladding material of composite insulator is silicone rubber, and the discarding will result in resources waste and vast expanses of land occupied (Figure 2). Moreover, the combustion disposal is inappropriate as its combustion gases are very contaminative and highly toxic. Therefore, it is an urgent and complicated problem to deal with the plenty of waste silicone rubber based composite insulators.

The same challenge for dealing with the waste tire rubber has existed since the development of the auto industry. Afterwards the problem deserves attention, and the recycling of waste tire rubber is chosen to reduce the adverse impact on the environment. The construction and concrete can be considered as one of the best alternatives to consume recycled rubber [7]. The studies on waste tire rubber mortar and concrete have been carried out since the 1990s. Owing to the low specific gravity of tire rubber particles, the apparent density of rubber mortar is less than plain mortar and decreases with the increase of the tire rubber content [8]. The compressive strength and flexural strength tests indicate that the tire rubber mortar and concrete have lower mechanical strength, but fine ductility [7,9,10,11,12,13]. The tire rubber mortar and concrete exhibit the superior thermal insulation in comparison with the plain ones due to higher air content and porosity [14,15]. According to previous durability research [16,17,18,19,20,21] it can be concluded that the tire rubber mortar and concrete possess improved durability to the aggressive environment, such as the resistance to freezing and thawing, permeability resistance, anti-carbonation and anti-penetration of chlorine.

The hydrophobic nature of tire rubber is liable to cause the weak bonding strength between tire rubber and cement paste, so a lot of research has been performed to enhance hydrophilicity through surface modification of tire rubber, and then improve the whole performance of tire rubber mortar and concrete [22,23,24]. Mohammadi et al. reported in 2016 that the tire rubber treated by NaOH could observably improve the concrete mechanical strength [25]. Rivas-Vázquez et al. in 2015 discovered that the tire rubber concrete mechanical strength increased after the tire rubber was treated with acetone [26]. The tire rubber was firstly oxidized with KMnO_4_ and then sulfonated with NaHSO_3_ in the research by He et al. in 2016, and the mechanical property of the tire rubber concrete was significantly improved [27]. Onuaguluchi reported in 2015 that the limestone power pre-coated tire rubber and silica fume could improve the mechanical strength of rubberized mortar [28].

The studies on tire rubber and modified tire rubber for mortar and concrete have already achieved many useful results, and the rubberized mortar and concrete have been applied in the engineering field. Nevertheless, the recycling process of the waste tire rubber is complex and of high cost [29] and the component system is complicated due to waste tire composed of rubber, tire cord (steel cord, nylon cord, etc.), carbon black, adhesive substance and so on. In contrast, the waste silicon rubber of composite insulators has a simple composition, consisting of the basis material of polydimethylsiloxane, which possesses the molecular chain structure comprising the Si–O main chain and –CH_3_ side group, and about 55 wt% aluminum hydroxide particle filler [1,30]. The silicone rubber, even the waste one, still has better abrasion resistance, preferable chemical resistance, more excellent high and low temperature resistance, and is less adverse to health and the environment, compared with waste tire rubber [31,32,33]. According to our knowledge, a rare study on the application of silicone rubber in mortar or concrete has been reported. In one study carried by Richardson et al. in 2012, the high silicone content rubber crumb of waste tire was added into concrete and only 0.6% rubber crumb content by weight could provide significant freeze-thaw protection [21]. Other slightly related studies were carried out on liquid-type silicone rubber used in polymer concretes [34,35]. Therefore the study on the application of waste silicone rubber in construction materials is necessary and significant. This paper attempts to explore the recycling approach and provide an application basis of the waste silicone rubber of the composite insulator in cement mortar. Firstly, the effect of silicone rubber on mortar mechanical strength was studied. Then the H_2_O_2_ and KOH were used to enhance the hydrophilicity of silicone rubber, and the mechanical strength between silicone rubber mortar and the modified ones was compared. The thermal conductivity of silicone rubber mortar was measured considering its great application value as a thermal insulation mortar. Besides, the potential utilization of silicone rubber mortar as noise reduction ground was investigated. This study can not only provide a useful reference for silicone rubber applying to thermal and noise insulation mortar, but also contributes to alleviating the adverse impact on environment brought by the waste composite insulator.

## 2. Materials and Methods

### 2.1. Materials

The cement is Ordinary Portland cement of Grade 42.5 (P.O 42.5) manufactured according to the Chinese Standard GB175-2007. The physical properties of the cement are presented in Table 1.

The fine aggregate is graded natural river sand, and its apparent density and fineness modulus are 2580 kg/m^3^ and 2.69, respectively.

**Figure 3:**
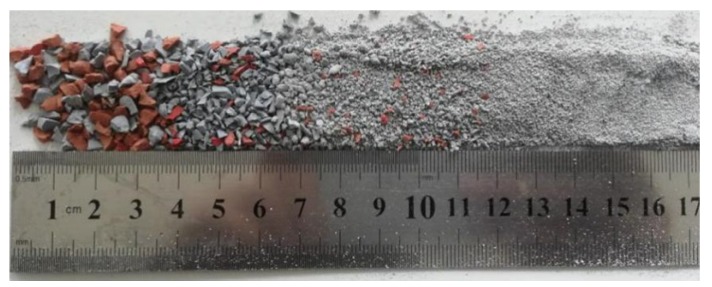
Silicone rubber particles.

The silicone rubber is peeled off from the reclaimed composite insulator, which are supplied by State Grid Henan Electric Power Company, China, and then broken to certain size particles (Figure 3). The apparent density and size distribution of the silicone rubber particles are 1436 kg/m^3^ and 5–50 mesh, respectively.

### 2.2. Silicone Rubber Modification

The modifiers for silicone rubber include H_2_O_2_ and KOH. In the H_2_O_2_ instance, the silicone rubber particles were soaked in H_2_O_2_ for 24 h at room temperature, and then dried naturally. In the KOH instance, firstly, the silicone rubber particles were soaked in saturated KOH solution for 24 h at room temperature. Then, the particles were washed by water several times until the pH value of the washing water approached neutral. Finally, the silicone rubber particles were dried in an oven at 60 °C to obtain the KOH modified silicone rubber particles.

### 2.3. Mix Proportions

The experiments included two stages. The first one was the exploratory test, in which the effect of silicone rubber on the mechanical strength was studied. The particle sizes of silicone rubber were 5, 10, 20 and 50 mesh. It was mixed into the mortar by equal volume substitution of sand, and the contents were 5%, 10%, 15%, 20% and 30%, respectively. The detailed mix proportions are shown in Table 2. The expanded test at the second stage was based on the results of the exploratory. The modified silicone rubbers were added in the mortar with particle size of 5 mesh and volume replacement rate of 5%, 15% and 30%, respectively. The mix proportions are listed in Table 3 in detail. The mechanical strength, thermal conductivity and noise reduction of the modified silicone rubber mortar were measured, and were compared with the original silicone rubber mortar. In the two stages tests, the mix proportion for the cement mortar was 1:0.5:3 (cement:water:fine aggregate).

### 2.4. Sample Preparation and Test Methods

#### 2.4.1. SEM and EDS Tests

A scanning electron microscope (SEM, Merlin Compact, Carl Zeiss NTS GmbH, Oberkochen, Germany) was used to observe the surface morphology of silicone rubber. The surface element content of silicone rubber was determined by an energy dispersive spectrometer (EDS).

#### 2.4.2. Contact Angle Test

A video-based optical contact angle measuring device (Shanghai Zhongchen Digital Technology Equipment Co. Ltd., Shanghai, China) was employed to detect the contact angle between silicone rubber (including original silicone rubber and the modified ones) and deionized water, in order to analyze the polarity change of the silicone rubber surface after modification.

#### 2.4.3. Sample Preparation

Silicone rubber, river sand, cement and water were weighed accurately according to the mix proportions. The mixing process was performed according to the Chinese Standard JGJ/T70-2009. All the molds were lubricated previously with mineral oil to prevent the mortar from adhering to the mold walls. For each mixture, the mortar specimens with the dimensions of 40 mm × 40 mm × 160 mm was fabricated to measure the compressive strength and flexural strength at 7 days and 28 days. The specimens for thermal conductivity and noise reduction tests were plate-shape with the size of 200 mm × 200 mm × 15 mm. All the mortar specimens were applied ambient curing for the first day. After demolding, they were cured to a certain curing period under the standard conditions with a temperature of 20 ± 2 °C and relative humidity of higher than 90%.

#### 2.4.4. Mechanical Strength Test

The compressive strength and flexural strength measurements were performed in accordance with ASTM C 348-02 and C 109-02 by a universal testing machine (WDW-20, Hengruijin Testing Machine Co., Ltd., Jinan, China), and the loading rates were 3 and 150 kN/min, respectively.

#### 2.4.5. Thermal Conductivity Test

To evaluate the effect of the silicone rubber and the modified one on the heat transfer ability of the cement mortars, the thermal conductivity test was carried out by means of a rapid thermal conductivity measurement system (Jiantong Environmental Technology Co., Beijing, China) with the temperature of 30 °C and the humidity of 47%. The mortar specimens were dried at 60 °C for 2 days before testing.

#### 2.4.6. Noise Reduction Test

**Figure 4:**
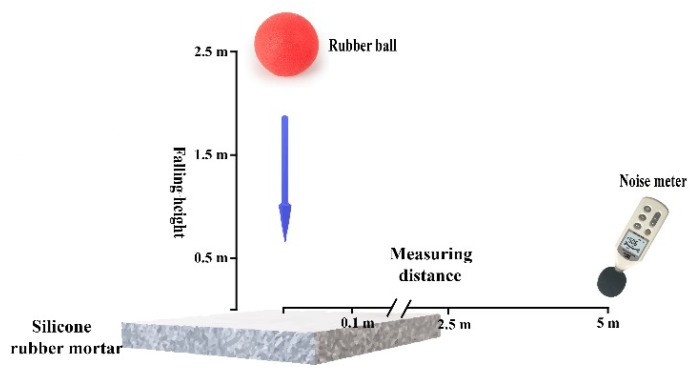
Self-made rubber ball impact test device.

A self-made rubber ball impact test device was used to measure the noise reduction of the mortar specimen as shown in Figure 4. The rubber ball fell freely from a specified height above the mortar plate (0.5, 1.5 and 2.5 m), and a noise meter (Sigma Technology Co., Ltd., Qingdao, China) was used to measure the noise level at different distances (0.1, 2.5 and 5.0 m). The rubber ball was 70.5 g and its diameter was 48.4 mm.

## 3. Results

### 3.1. Characterization of Silicone Rubber

#### 3.1.1. SEM and EDS

**Figure 5:**
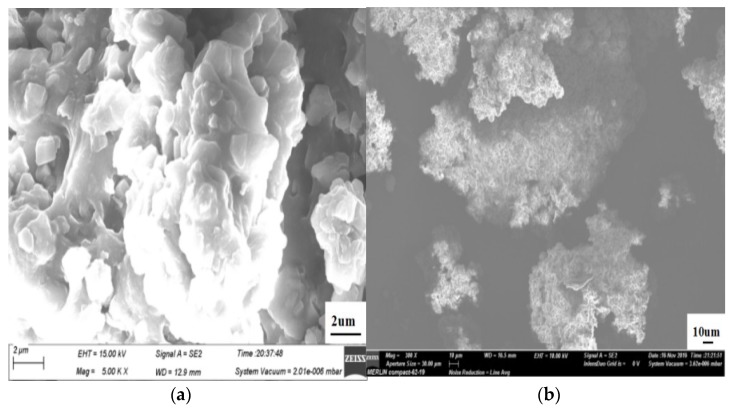
SEM images of silicone rubber powder. (**a**) 5000× magnification; (**b**) 300× magnification.

**Figure 6:**
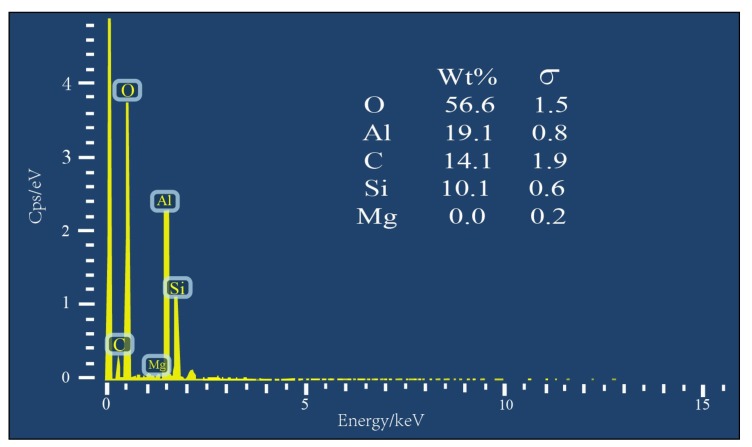
Energy dispersive spectrometer (EDS) spectrum of silicone rubber powder.

The SEM images (Figure 5) show that the silicone rubber powder was uneven in surface and irregular in shape, and there was no obvious agglomeration among them. The EDS spectrum of silicone rubber (Figure 6) shows that the main elements of silicone rubber surface included C, O, Al and Si. Among them, C and Si were mainly from the polydimethylsiloxane matrix of the silicone rubber, and Al and O stemmed mainly from the aluminum hydroxide filler.

#### 3.1.2. Contact Angle

**Figure 7:**
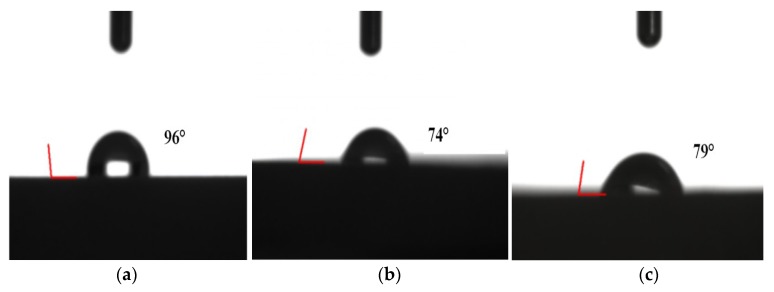
Testing photographs of the water contact angle of silicone rubber. (**a**) Original; (**b**) H_2_O_2_ treated; (**c**) KOH treated.

In order to analyze the surface property change of the modified silicone rubber, as exhibited in Figure 7 the water contact angles of original silicone rubber (96°), H_2_O_2_ modified silicone rubber (74°) and KOH modified silicone rubber (79°) were measured, respectively. That is, through the modification, the surface property of silicone rubber changed from hydrophobic to hydrophilic as a small number of the hydroxyl groups exist on its surface after modification by H_2_O_2_ or KOH [27,36].

### 3.2. Mechanical Strength

#### 3.2.1. Effect of Silicone Rubber on the Mechanical Strength of Mortar

**Figure 8:**
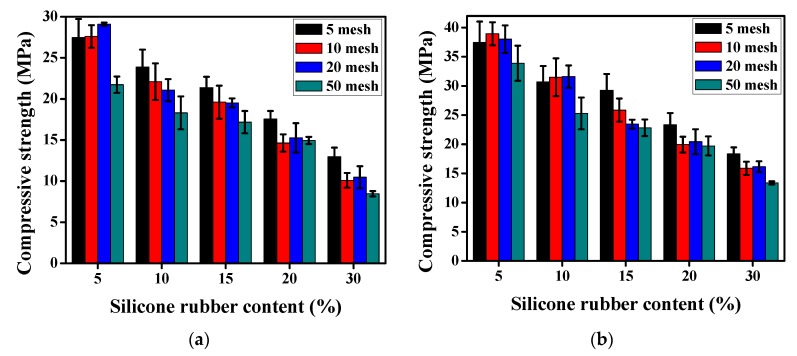
The compressive strength of the silicone rubber mortar. (**a**) 7-day; (**b**) 28-day.

The 7-day and 28-day compressive strengths of the plain mortar were 30.2 MPa and 46.68 MPa. The compressive strengths variations of 7-day and 28-day on account of the size and the content changes of silicone rubber particles are illustrated by Figure 8a,b, respectively. Similar to the tire rubber mortar, the compressive strength decreased with the silicone rubber content increasing. Take the 10 mesh silicone rubber for instance, as the content improved from 5% to 30%, the 7-day compressive strength of mortar decreased from 27.6 MPa to 10.1 MPa and the 28-day’ decreased from 38.95 MPa to 15.88 MPa. Compared with the plain mortar, the 7-day compressive strength dropped by 8.61% and 66.56%, and the 28-day’ dropped by 16.56% and 65.98%, respectively. Within the ranges of this study, the compressive strength tended to rise slightly with the increase of silicone rubber size. Moreover, they all were below the compressive strength of the plain mortar.

**Figure 9:**
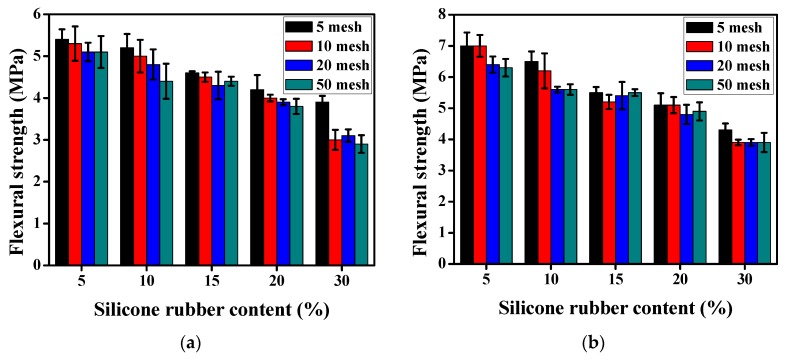
The flexural strength of silicone rubber mortar. (**a**) 7-day; (**b**) 28-day.

The 7-day flexural strength of the plain mortar was 5.8 MPa and the 28-day’ was 7.3 MPa. Figure 9a,b show the effect of silicone rubber on the 7-day and 28-day flexural strength of the mortar. As can be seen, the flexural strength decreased gradually with the increase of silicone rubber content regardless of the particle size. Taking 10 mesh silicone rubber powder as an example, with the silicone rubber content increase from 5% to 30%, the 7-day flexural strength decrease from 5.3 MPa to 3 MPa and the 28-day’ decrease from 7 MPa to 3.9 MPa. In comparison with plain mortar, the 7-day flexural strength declined by 8.62% and 48.28%, and the 28-day’ declined by 4.11% and 46.58%, respectively. In addition, similar to the compressive strength, a less obvious increasing trend for the flexural strength can be observed accompanying the increase of particle size.

The reasons of reduction in the mechanical strength of the silicone rubber mortar are related to the properties of silicone rubber [12,16,37]. Compared with the fine aggregates like river sand, the stiffness of the silicone rubber is low. Hence the presence of the silicone rubber in mortar reduces the stiffness and lowers its load bearing capacity. In addition, the silicone rubber is hydrophobic, which causes the weak bond between silicone rubber and cement paste. Therefore, the degree of weakness increases, which leads to the declined strength. Previous research has shown that the rubber has the air-entraining ability in the process of adding and stirring in the cement paste as its rough surface morphology and hydrophobic surface [38,39]. Based on it, the mode pore diameter and the total porosity in the mortar gradually increases as a result of the improvement of the rubber particle content. The pore will become harmful even disastrous pores as pore diameter larger than 100 nm [39], bringing about a harmful effect on mechanical strength. The more silicone rubber the mortar contains, the lower the mortar strength is. As for the elevating trend of strength accompanying the increase of particle size, it can be explained that the interfacial transition zone between the cement paste and the silicone rubber particles reduces as the bigger particle possessing smaller total surface area.

**Figure 10:**
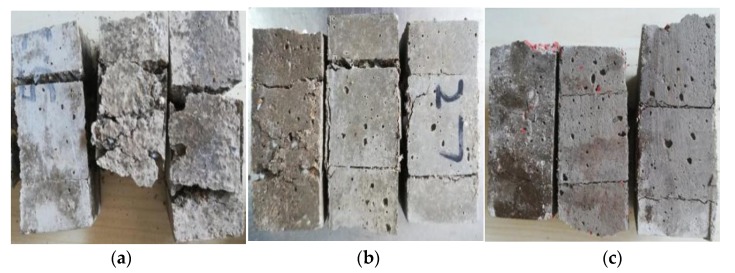
Failure modes of the mortar specimen in the compressive test, (**a**) plain (**b**) 5 mesh silicone rubber, 15% content, and (**c**) 5 mesh silicone rubber, 30% content.

The failure modes of the mortar in the compressive test are shown in Figure 10. The plain mortar specimens exhibited brittle failure (Figure 10a). When the content of silicone rubber was 5%, the brittle failure obviously reduced (Figure 10b). There was no brittle failure of the 30% silicone rubber mortar under compressive loading (Figure 10c). This phenomenon proved that the addition of silicone rubber could improve the toughness of mortar, coinciding with the previous studies [10,27].

#### 3.2.2. Effect of the Modified Silicone Rubber on the Mechanical Strength of Mortar

**Figure 11:**
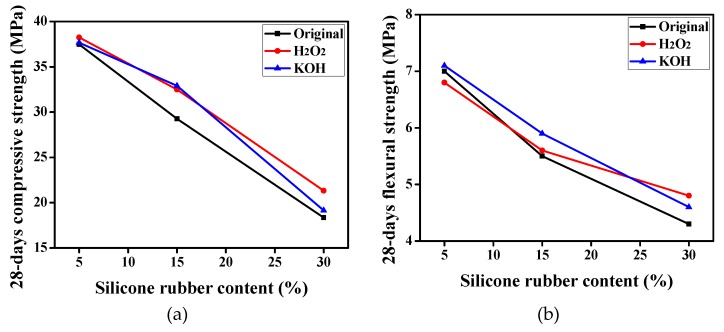
The 28-day mechanical strength of modified silicone rubber mortar. (**a**) Compressive strength; (**b**) Flexural strength.

The effects of H_2_O_2_ and KOH modified silicone rubbers on the 28-day mechanical strength of mortar are displayed in Figure 11. The silicone rubber treated by H_2_O_2_ or KOH solution has an enhancing effect compared with the original silicone rubber, especially the compressive strength. For example, when the content of silicone rubber was 15%, compared with original rubber, the 28-day compressive strengths treated by H_2_O_2_ and KOH improved by 10.56% and 12.44% respectively, and the 28-day flexural strengths improved by 1.82% and 7.27% respectively.

The possible reasons for this effect are shown as follows. H_2_O_2_ can remove the impurities on the surface of silicone rubber and improve its surface hydrophilicity, which ameliorates the interface between silicone rubber and cement paste, further increases the mechanical strength of mortar. Similarly, after treatment with KOH solution the hydrophilic property of the silicone rubber increases according to the contact angle result. In addition, the KOH can provide a weak alkali condition around the silicone rubber particles, which is conducive to the cement hydration around it, so a higher hydration degree and lower porosity of transition zone can be realized [36], by virtue of which the mechanical strength of silicone rubber mortar has an obvious improvement.

### 3.3. Thermal Insulation

#### 3.3.1. Thermal Conductivity

In order to fulfill the increasing demands of indoor comfort and energy efficiency requirements of building, the innovative material with high thermal insulation, for example, the rubber mortar has attracted more and more attention. The thermal conductivity is an important characteristic to evaluate the thermal insulation performance.

**Figure 12:**
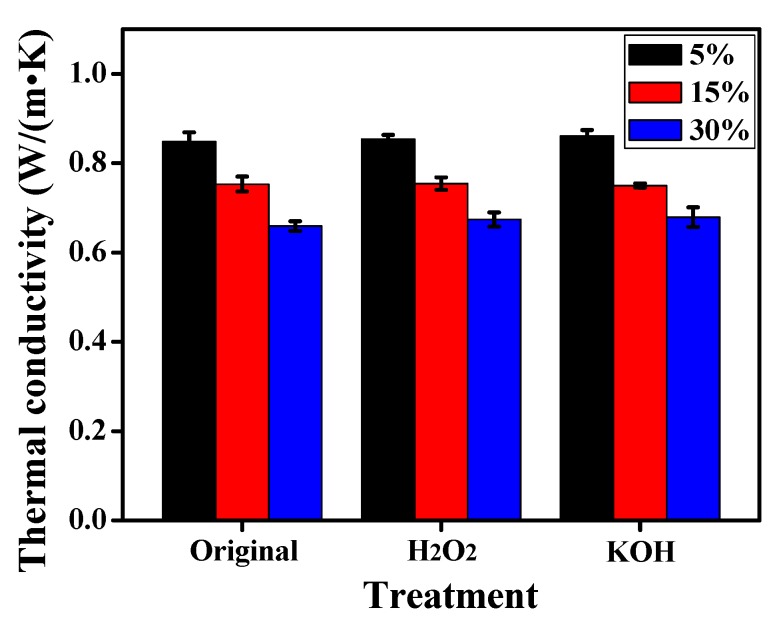
Thermal conductivity of the original and modified silicone rubber mortar.

In the study, the thermal conductivity of plain mortar was 1.09 W/(m·K). As shown in Figure 12, when the silicone rubber content was 5%, 15% and 30%, the thermal conductivity was approximately 77.71%, 68.81% and 60.64% of the plain mortar, respectively. The thermal conductivity of mortar declines as a consequence of the increase porosity due to the heat preservation of air in the pores. As hereinbefore discussed, the air-entraining ability of rubber leads to the increase of the total porosity, including the interface porosity and the porosity in cement paste [39]. Hence the thermal conductivity decreases accompanying the increase of silicon rubber content. Moreover, pure silicon rubber usually has poor thermal conductivities of 0.165 W/m·K [40], which is another factor for the better thermal insulation of mortar [41].

In addition, it can also be seen that the modified silicon rubber mortar showed a slightly higher thermal conductivity compared with the original one. For instance, the mortars containing 30% modified silicone rubber by H_2_O_2_ and KOH, the thermal conductivities enhanced by 1.21% and 2.42%, respectively. This also shows that the compactness of KOH modifying silicon rubber mortar was slightly higher than the H_2_O_2_ modifying one, meaning the former possessing lower porosity, and this was in agreement with the mechanical strength results, also known as mortar containing 30% modified silicone rubber by H_2_O_2_ and KOH exhibiting the 16.24% and 4.36% higher compressive strength, and the 10.42% and 6.52% higher flexural strength, respectively, in Section 3.2.2. The air-entraining ability is on account of the rough surface morphology and hydrophobic surface. Whereas, the rough surface morphology hardly changes through the modification treatment, so there is little change of the porosity especially in cement paste, resulting in the thermal conductivities to be quite close between the original silicone rubber mortar and the modified ones.

#### 3.3.2. Dry Apparent Density

**Figure 13:**
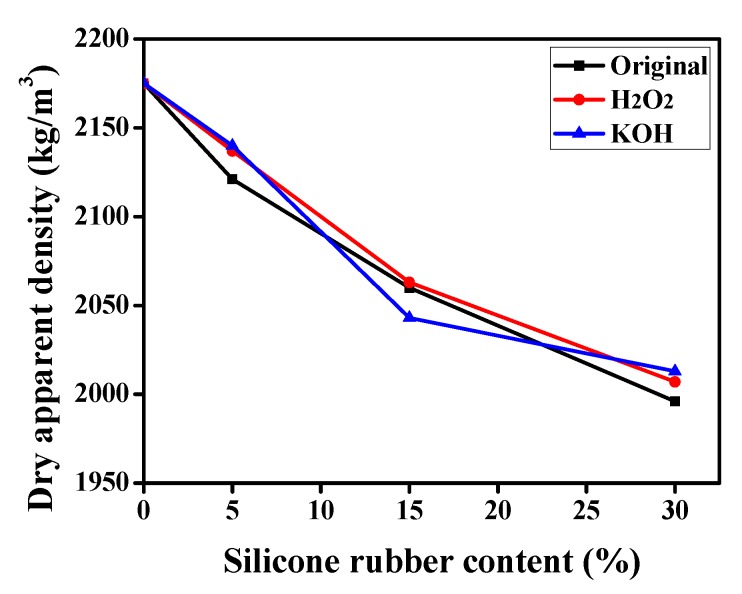
Dry apparent density of the original and modified silicone rubber mortar.

The dry apparent density of mortar is of great significance to the cost and convenience of the construction, especially mortar pouring and repairing. It is affected by many factors, such as material density, water cement ratio and air content. The apparent density of silicone rubber is 1436 kg/m^3^, which is quite lesser than 2570 kg/m^3^ of fine aggregates of river sand. As can be seen in Figure 13, the dry apparent density of mortar was from 2175 to 1996 kg/m^3^, which decreased with the increasing percent of the replacement of river sand by silicone rubber. One of the reasons for the reduction in mortar density is due to the lesser apparent density of silicone rubber, besides, another reason lies in the silicone rubber entrapping air effect and subsequent higher porosity [13,38,39,42]. However, the modification of silicone rubber had no obvious effect, which was consistent with the thermal conductivity.

**Figure 14:**
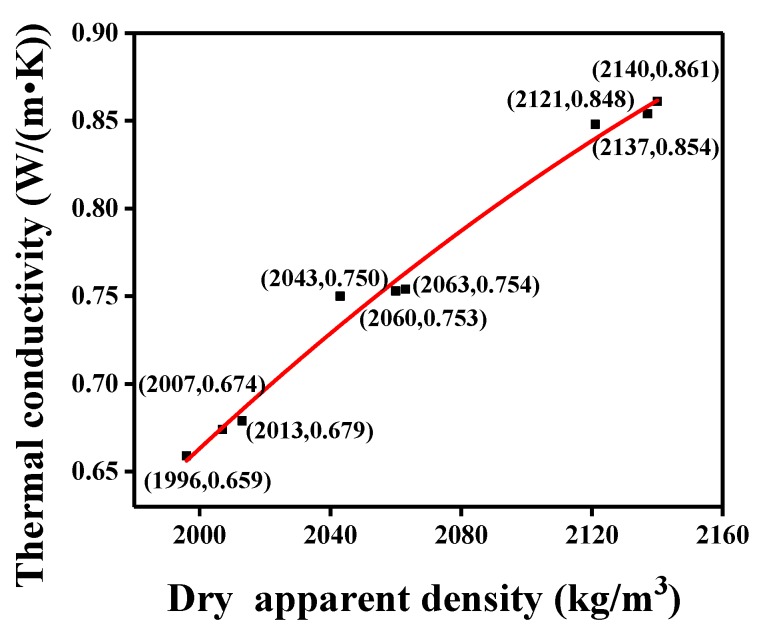
Fitted curve between thermal conductivity and dry apparent density.

With the silicone rubber content increased, the thermal conductivity of silicone rubber mortar showed a similar variation trend with the dry apparent density according to the test results. As shown by the points in Figure 14, the thermal conductivity increased with the dry apparent density and a good correlation was recognizable. Therefore, the functional relationship between them was established as:

λ = −2.22 × 10^−6^*ρ*^2^ + 0.0106*ρ* − 11.6729 (R^2^ = 0.9862), (1)

where *ρ* is the dry apparent density of the silicone rubber mortar, kg/m^3^ and *λ* is the thermal conductivity of silicone rubber mortar, W/(m·K).

### 3.4. Noise Reduction

**Figure 15:**
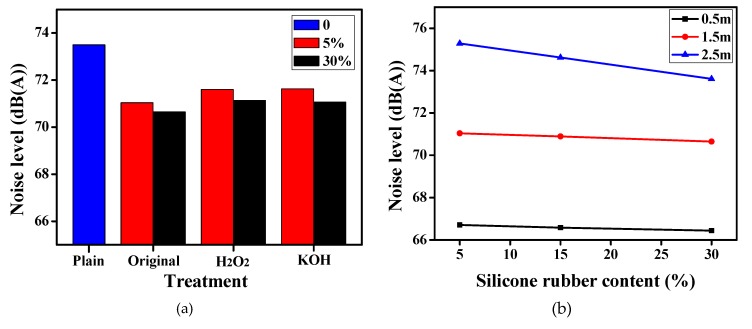

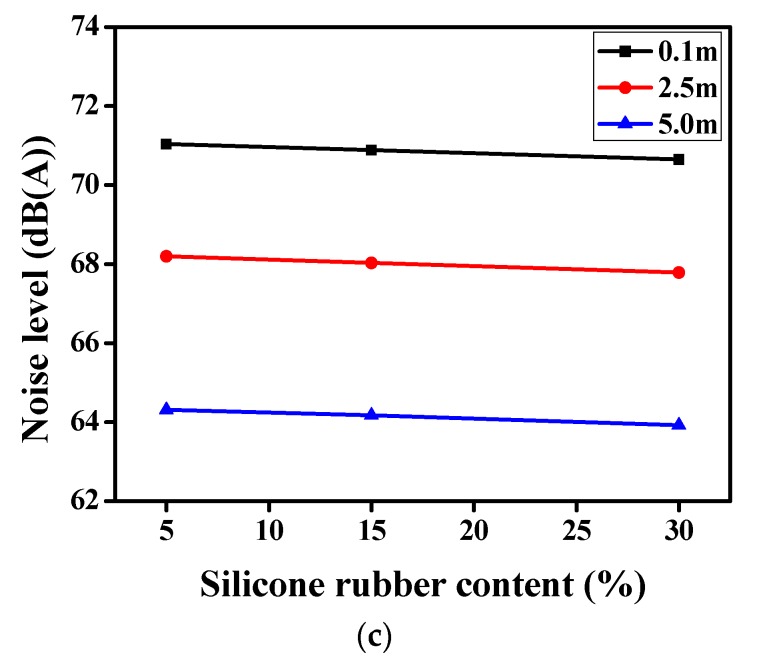
Effect of test conditions on the noise level of silicone rubber mortar. (**a**) Modification method (measuring distance: 0.1 m; falling height: 1.5 m); (**b**) Falling height (original silicon rubber; measuring distance: 0.1m); (**c**) Measuring distance (original silicon rubber; falling height: 1.5 m).

The noise reduction of the mortar is an essential performance when it is used for indoor ground. In this study, the rubber ball impact method was used to simulate the noise generated by hitting the ground of the sole. As shown in Figure 15a,b, the noise level of the mortar decreased with the increase of silicone rubber content. For example, it was 73.5 dB (A) for the plain mortar with 1.5 m falling height, when 5% and 30% original silicone rubber was added, it reduced to 71.04 dB (A) and 70.65 dB (A), respectively. It also can be seen that the noise level for the mortar containing modified silicon rubber was slightly higher than the original ones, which might be connected with the little smaller porosity. Besides, the noise level increased with the increase of falling height from 0.5 m to 2.5 m, and the noise reduction brought by silicone rubber also became more significant at the same time. The effect of measuring distance on the noise level was exhibited in Figure 15c with the falling height of 1.5 m. The curves of different measuring distances were almost parallel straight lines with fixed spaces, and the noise level of 0.1 m measuring distance was about 2.86 dB (A) and 6.73 dB (A) higher than that of 2.5 m and 5 m, respectively. Besides, as the silicone rubber content increased by 5% to 30%, the noise reduction was about 0.4 dB (A).

The principle of the noise reduction improvement is similar to the air-entraining effect of the silicone rubber. The porosity of mortar increased along with the addition amount of silicone rubber as discussed above, which could increase the frequency of acoustic reflection caused by the rubber ball impact, achieving the energy dissipation. At the meantime, the elastic modulus of mortar reduced after adding elastic silicone rubber, which increased the adaptability of stress diffusion and stress absorption, and consumed more sound energy.

## 4. Conclusions

In this paper a novel approach to reuse waste silicone rubber of composite insulator applying to the cement mortar was developed. In addition, a valid attempt to modify the silicone rubber surface by KOH and H_2_O_2_ was carried out.

The silicone rubber was proved to be a good substitute for fine aggregate to enhance the mortar toughness according to the failure modes. The compressive strength and flexural strength decreased with the silicone rubber content increasing. However the strength of modified silicone rubber mortar improved compared with the original one.

As the silicone rubber increased, the mortar porosity went up resulting from the air-entraining ability and the expanding loose interfacial transition zone, and the thermal insulation and noise reduction performances of the silicone rubber mortar enhanced as a result. Moreover, the low thermal conductivity of the silicone rubber was favorable for the thermal insulation of mortar, and the high elasticity of the silicone rubber was beneficial for the noise reduction of mortar. The thermal conductivity of 30% silicone rubber content mortar remarkably decreased to 60.64% of the plain mortar. Furthermore, as to the silicone rubber mortar there was a good correlation between thermal conductivity and dry apparent density, and a quadratic function was obtained to describe the relationship. A self-made rubber ball impact test device was used to measure the noise reduction when the measuring distance was 0.1 m and falling height was 1.5 m, the mortar noise level reduced by 2.46 dB (A) and 2.85 dB (A) with the 5% and 30% silicone rubber content, respectively.

This study not only contributes to reuse waste silicone rubber in thermal and noise insulation mortar, expanding the cement mortar application field, but also helpfully reduces solid waste, providing valuable reference for environment protection. Moreover, further research on mechanical strength improvement of silicone rubber mortar, as well as silicon rubber addition to concrete will be carried out for the next stage.

## Figures and Tables

**Table 1:** Physical properties of cement.

Property	Specific Density (kg/m^3^)	Normal Consistency (%)	Initial Setting Time (min)	Final Setting Time (min)	Compressive Strength (MPa)		Flexural Strength (MPa)	
					7 days	28 days	7 days	28 days
Cement	3100	28.35	235	305	30.2	46.7	5.8	7.3

**Table 2:** The detailed mix proportions of mortar in the first stage test (kg/m^3^).

Description of Mortar	Cement	Water	River Sand	Silicone Rubber
Plain	503.68	251.84	1511.04	0
Replacing 5%; 5 mesh	503.68	251.84	1435.49	42.05
Replacing 10%; 5 mesh	503.68	251.84	1359.94	84.10
Replacing 15%; 5 mesh	503.68	251.84	1284.38	126.15
Replacing 20%; 5 mesh	503.68	251.84	1208.83	168.20
Replacing 30%; 5 mesh	503.68	251.84	1057.73	252.30
Replacing 5%; 10 mesh	503.68	251.84	1435.49	42.05
Replacing 10%; 10 mesh	503.68	251.84	1359.94	84.10
Replacing 15%; 10 mesh	503.68	251.84	1284.38	126.15
Replacing 20%; 10 mesh	503.68	251.84	1208.83	168.20
Replacing 30%; 10 mesh	503.68	251.84	1057.73	252.30
Replacing 5%; 20 mesh	503.68	251.84	1435.49	42.05
Replacing 10%; 20 mesh	503.68	251.84	1359.94	84.10
Replacing 15%; 20 mesh	503.68	251.84	1284.38	126.15
Replacing 20%; 20 mesh	503.68	251.84	1208.83	168.20
Replacing 30%; 20 mesh	503.68	251.84	1057.73	252.30
Replacing 5%; 50 mesh	503.68	251.84	1435.49	42.05
Replacing 10%; 50 mesh	503.68	251.84	1359.94	84.10
Replacing 15%; 50 mesh	503.68	251.84	1284.38	126.15
Replacing 20%; 50 mesh	503.68	251.84	1208.83	168.20
Replacing 30%; 50 mesh	503.68	251.84	1057.73	252.30

**Table 3:** The detailed mix proportions of mortar in the second stage test (kg/m^3^).

Description of Mortar	Cement	Water	River Sand	Silicone Rubber
Replacing 5%; 5 mesh	503.68	251.84	1435.49	42.05
Replacing 15%; 5 mesh	503.68	251.84	1284.38	126.15
Replacing 30%; 5 mesh	503.68	251.84	1057.73	252.30
Replacing 5%; 5 mesh; KOH treated	503.68	251.84	1435.49	42.05
Replacing 15%; 5 mesh; KOH treated	503.68	251.84	1284.38	126.15
Replacing 30%; 5 mesh; KOH treated	503.68	251.84	1057.73	252.30
Replacing 5%; 5 mesh; H_2_O_2_ treated	503.68	251.84	1435.49	42.05
Replacing 15%; 5 mesh; H_2_O_2_ treated	503.68	251.84	1284.38	126.15
Replacing 30%; 5 mesh; H_2_O_2_ treated	503.68	251.84	1057.73	252.30

## References

[1] Verma A.R., Reddy B.S. (2017). Accelerated aging studies of silicon-rubber based polymeric insulators used for HV transmission lines. Polym. Test..

[2] Buontempo R.C., Dellallibera A.A., Costa E.C.M., Pissolato J., Mello D.R.D., Mei L.H.I. (2016). Electrical assessment of commercial 6.0-kV HTV silicone rubber for power insulation. Measurement.

[3] Liang X.D., Gao Y.F., Wang J.F., Li S.H. (2016). Rapid development of silicone rubber composite insulator in China. High Volt. Eng..

[4] Zhang G.J., Zhao L., Zhou R.D., Shen W.W., Liang X.D. (2016). Review on aging characterization and evaluation of silicon rubber composite insulator. High Volt. Appar..

[5] Cardoso R., Balestro A.C., Dellallibera A., Costa E.C.M., Angelini J.M.G., Mei L.H.I. (2014). Silicone insulators of power transmission lines with a variable inorganic load concentration: Electrical and physiochemical analyses. Measurement.

[6] Arshad, Nekahi A., Mcmeekin S.G., Farzaneh M. (2017). Measurement of surface resistance of silicone rubber sheets under polluted and dry band conditions. Electr. Eng..

[7] Jalal M., Nassir N., Jalal H. (2019). Waste tire rubber and pozzolans in concrete a trade-off between cleaner production and mechanical properties in a greener concrete. J. Clean. Prod..

[8] Oikonomou N., Mavridou S. (2009). Improvement of chloride ion penetration resistance in cement mortars modified with rubber from worn automobile tires. Cement Concrete Compos..

[9] Batayneh M.K., Marie I., Asi I. (2008). Promoting the use of crumb rubber concrete in developing countries. Waste Manag..

[10] Al-Tayeb M.M., Bakar B.H.A., Akil H.M., Ismail H. (2013). Performance of rubberized and hybrid rubberized concrete structures under static and impact load conditions. Exp. Mech..

[11] Ganesan N., Raj J.B., Shashikala A.P. (2013). Flexural fatigue behavior of self compacting rubberized concrete. Constr. Build. Mater..

[12] Youssf O., Hassanli R., Mills J.E. (2017). Mechanical performance of FRP-confined and unconfined crumb rubber concrete containing high rubber content. J. Build. Eng..

[13] Abdelaleem B.H., Ismail M.K., Hassan A.A.A. (2018). The combined effect of crumb rubber and synthetic fibers on impact resistance of self-consolidating concrete. Constr. Build. Mater..

[14] Sukontasukkul P. (2009). Use of crumb rubber to improve thermal and sound properties of pre-cast concrete panel. Constr. Build. Mater..

[15] Kader M.M.A., Abdel-Wehab S.M., Helal M.A., Hassan H.H. (2012). Evaluation of thermal insulation and mechanical properties of waste rubber/natural rubber composite. HBRC J..

[16] Sofi A. (2018). Effect of waste tyre rubber on mechanical and durability properties of concrete—A review. Ain Shams Eng. J..

[17] Gesoğlu M., Güneyisi E., Khoshnaw G., İpek S. (2014). Abrasion and freezing–thawing resistance of pervious concretes containing waste rubbers. Constr. Build. Mater..

[18] Topçu I.B., Demir A. (2007). Durability of Rubberized Mortar and Concrete. J. Mater. Civ. Eng..

[19] Bravo M., Brito J.D. (2012). Concrete made with used tyre aggregate: Durability-related performance. J. Clean. Prod..

[20] Kardos A.J., Durham S.A. (2015). Strength, durability, and environmental properties of concrete utilizing recycled tire particles for pavement applications. Constr. Build. Mater..

[21] Chou L.H., Lin C.N., Lu C.K., Lee C.H., Lee M.T. (2010). Improving rubber concrete by waste organic sulfur compounds. Waste Manag. Res..

[22] Albano C., Camacho N., Reyes J., Feliu J.L., Hernández M. (2005). Influence of scrap rubber addition to Portland I concrete composites: Destructive and non-destructive testing. Compos. Struct..

[23] Marques A.C., Akasaki J.L., Trigo A.P.M., Marques M.L. (2008). Influence of the surface treatment of tire rubber residues added in mortars. Rev. IBRACON Estrut. Mater..

[24] Mohammadi I., Khabbaz H., Vessalas K. (2016). Enhancing mechanical performance of rubberised concrete pavements with sodium hydroxide treatment. Mater. Struct..

[25] Rivas-Vázquez L.P., Suárez-Orduña R., Hernández-Torres J., Aquino-Bolaños E. (2015). Effect of the surface treatment of recycled rubber on the mechanical strength of composite concrete/rubber. Mater. Struct..

[26] He L., Ma Y., Liu Q., Mu Y. (2016). Surface modification of crumb rubber and its influence on the mechanical properties of rubber-cement concrete. Constr. Build. Mater..

[27] Onuaguluchi O. (2015). Effects of surface pre-coating and silica fume on crumb rubber-cement matrix interface and cement mortar properties. J. Clean. Prod..

[28] Siddika A., Mamun M.A.A., Alyousef R., Amran Y.H.M., Aslani F., Alabduljabbar H. (2019). Properties and utilizations of waste tire rubber in concrete: A review. Constr. Build. Mater..

[29] Xue Y., Li X., Zhang D., Wang H., Chen Y., Chen Y. (2018). Comparison of ATH and SiO_2_ fillers filled silicone rubber composites for HTV insulators. Compos. Sci. Technol..

[30] Arshad, Nekahi A., Mcmeekin S.G., Farzaneh M. (2017). Effect of pollution severity and dry band location on the flashover characteristics of silicone rubber surfaces. Electr. Eng..

[31] Wang C., Li T., Tu Y., Yuan Z., Li R., Zhang F., Gong B. (2016). Heating phenomenon in unclean composite insulators. Eng. Fail. Anal..

[32] Verma A.R., Reddy B.S. (2018). Aging studies on polymeric insulators under DC stress with controlled climatic conditions. Polym. Test..

[33] Richardson A.E., Coventry K.A., Ward G. (2012). Freeze/thaw protection of concrete with optimum rubber crumb content. J. Clean. Prod..

[34] Jung K.C., Roh I.T., Chang S.H. (2015). Thermal behavior and performance evaluation of epoxy-based polymer concretes containing silicone rubber for use as runway repair materials. Compos. Struct..

[35] Roh I.T., Jung K.C., Chang S.H., Cho Y.H. (2015). Characterization of compliant polymer concretes for rapid repair of runways. Constr. Build. Mater..

[36] Si R., Guo S., Dai Q. (2017). Durability performance of rubberized mortar and concrete with NaOH-Solution treated rubber particles. Constr. Build. Mater..

[37] Poon C.S., Shui Z.H., Lam L. (2004). Effect of microstructure of ITZ on compressive strength of concrete prepared with recycled aggregates. Constr. Build. Mater..

[38] Nehdi M., Khan A., Nehdi M. (2001). Cementitious composites containing recycled tire rubber: An overview of engineering properties and potential applications. Cement Concrete Aggreg..

[39] Yang L.H., Zhu H., Zhang Y.M. (2011). Effect of crumb rubber on pore structure of cement mortar. J. Tianjin Univ..

[40] Mu Q., Feng S., Diao G. (2007). Thermal conductivity of silicone rubber filled with ZnO. Polym. Compos..

[41] Wongsa A., Sata V., Nematollahi B., Sanjayan J., Chindaprasirt P. (2018). Mechanical and thermal properties of lightweight geopolymer mortar incorporating crumb rubber. J. Clean. Prod..

[42] Jafari K., Toufigh V. (2017). Experimental and analytical evaluation of rubberized polymer concrete. Constr. Build. Mater..

